# Supplementary material for: Spatially heterogeneous dynamics in a metallic glass forming liquid imaged by electron correlation microscopy
Source: Nat Commun. 2018 Mar 19;9:1129. doi: 10.1038/s41467-018-03604-2 (PMC5859095; doi:10.1038/s41467-018-03604-2)
Supplement: Supplementary file 1 — Description of Additional Supplementary Files(PDF 222 kb) [file 41467_2018_3604_MOESM1_ESM.pdf]

Description of Additional Supplementary Files

File Name: Supplementary Movie 1
Description: Example raw ECM data series Dark-field image series of a BMG nanowire held at 523 K. The series consists of 4000 images acquired. Playback is at 16 frames per second, but data were acquired at 10 frames per second.

File Name: Supplementary Movie 2
Description: Example ECM data series after alignment The same image series as in Supplementary Movie 1 after rigid image alignment to remove spatial drift of the sample. Playback is at the same rate as Supplementary Movie 1.

File Name: Supplementary Movie 3
Description: Fluctuating spatial domains A movie showing the time evolution of spatially heterogeneous dynamics at 523 K. A quadruple-length image series was analyzed using a “sliding window” to obtain a movie of τ as a function of time and space. Playback is at 16 frames per second, but data were acquired at 10 frames per second.
