# Supplementary material for: Spatially heterogeneous dynamics in a metallic glass forming liquid imaged by electron correlation microscopy
Source: Nat Commun. 2018 Mar 19;9:1129. doi: 10.1038/s41467-018-03604-2 (PMC5859095; doi:10.1038/s41467-018-03604-2)
Supplement: Supplementary file 6 — Supplementary Information(PDF 1857 kb) [file 41467_2018_3604_MOESM6_ESM.pdf]

Spatially Heterogeneous Dynamics in a Metallic Glass Forming Liquid Imaged by Electron Correlation Microscopy

Zhang *et al.*

Supplementary Figures

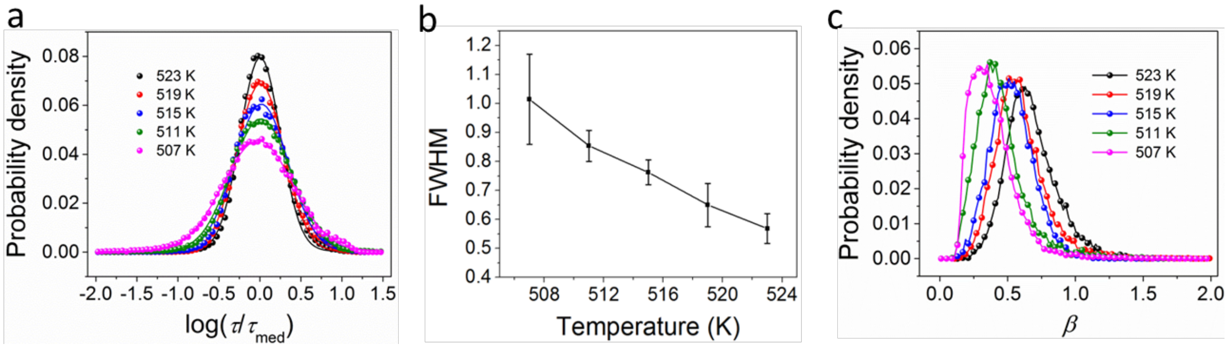

Supplementary Figure 1. (a) The distributions of relaxation time normalized by the median values in log scale at all five temperatures. The solid lines are the fit. (b) The full width of half maximum acquired from the fit in (a). The error is calculated from the standard deviation of the mean from five different data sets. (c) Distributions of β shift to lower value with decreasing temperature. The error is calculated by the standard deviation of the mean from four data sets.

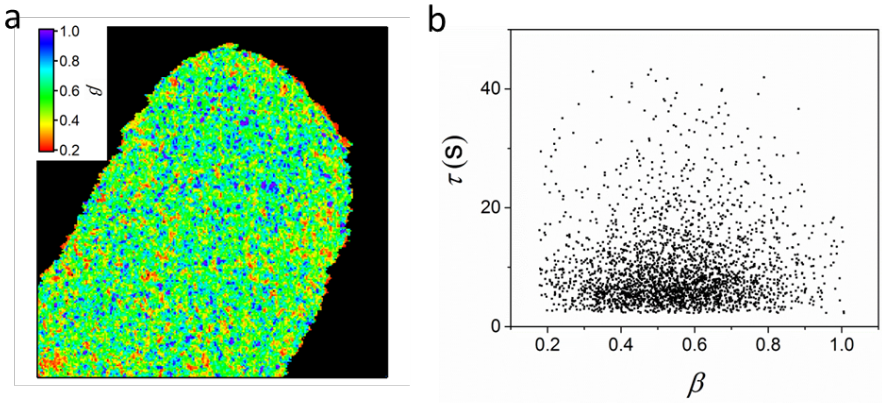

Supplementary Figure 2. (a) Spatial map of stretching exponent β for the same data set as the τ map in Fig. 2a at 523 K. (b) τ vs β for all the pixels in the map. There is no strong correlation between τ and β in this data set, and the parallel graphs at other temperatures are similar.

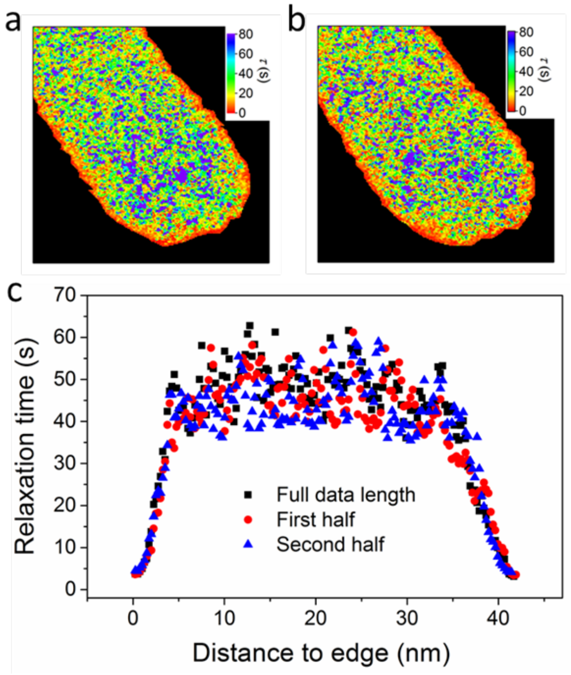

Supplementary Figure 3. Spatial map of relaxation time analyzed from (a) first 1000 s (b) second 1000 s at 519 K. The time per frame is 0.25 s. (c) The average relaxation time from free surface to inner bulk from first half of the data, the second half of the data and the full length of the data.

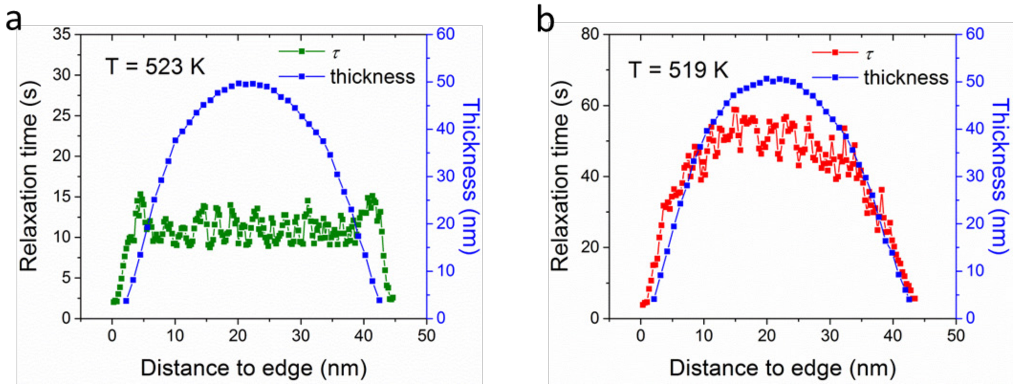

Supplementary Figure 4. The thickness dependence of relaxation time from round nanowires at temperature (a) 523 K (b) 519 K.

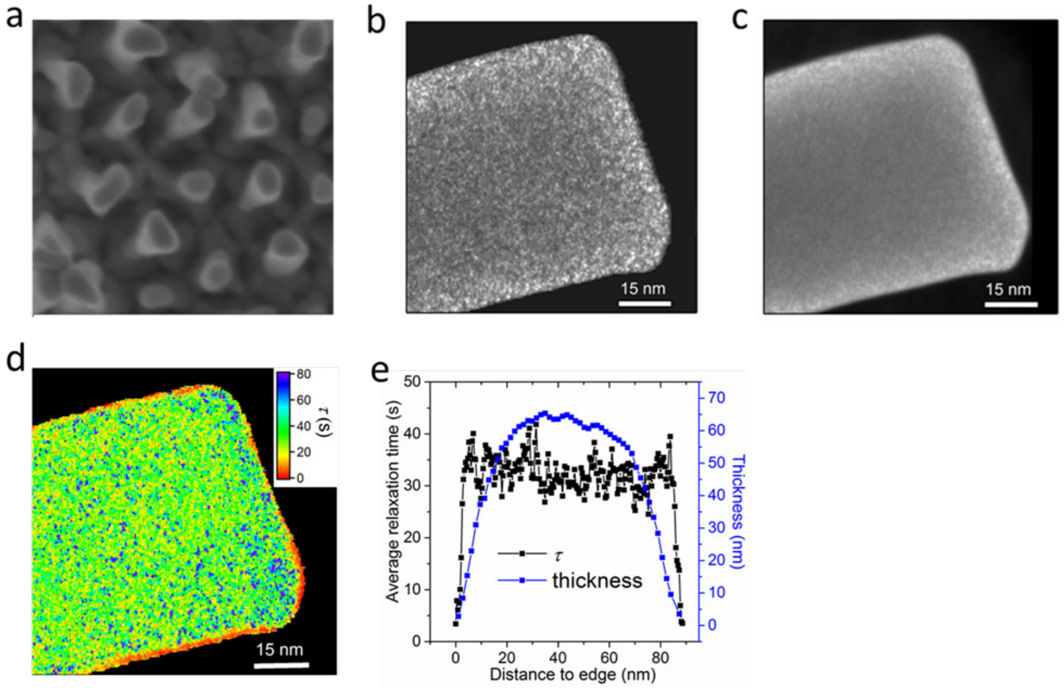

Supplementary Figure 5. (a) SEM image of nanowires attached to the substrate, viewed from the top down. (b) A snapshot of dark field image movie for a rectangular nanowire at T = 519 K. (c) The time-averaged dark field image taken from the same data series in (b). (d) The spatial map of relaxation time from the same data. (e) A profile of relaxation time and thickness across the wire.

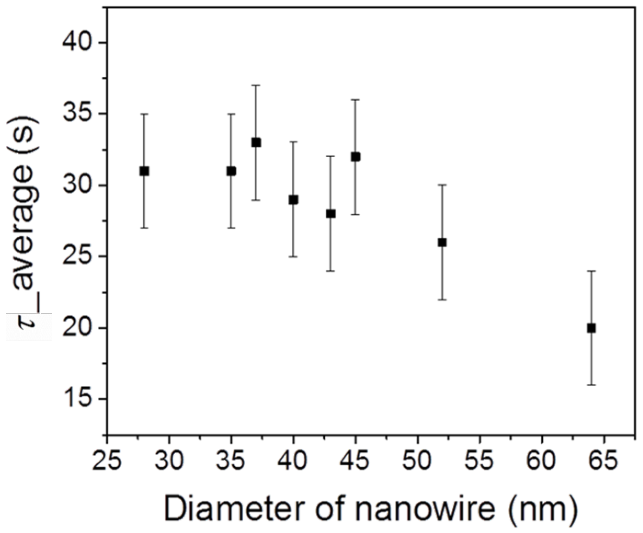

Supplementary Figure 6. Average relaxation time from inner bulk of nanowire as a function of diameter of nanowire. The uncertainty is calculated by the standard deviation of the mean from five different measurements.

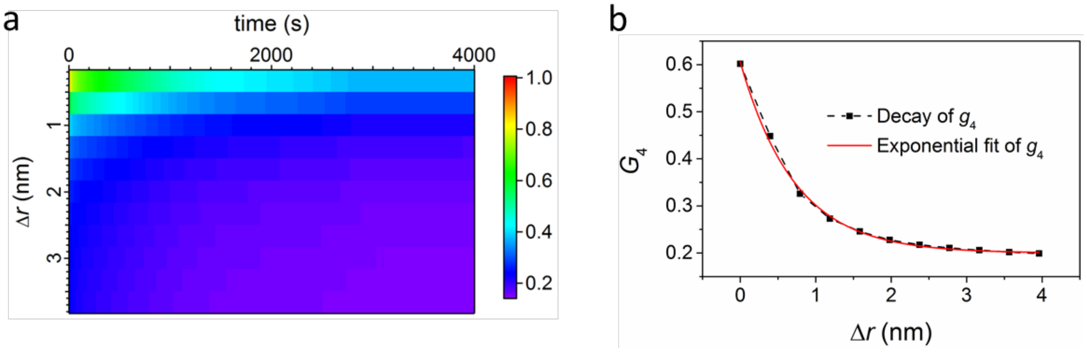

Supplementary Figure 7. (a) The normalized four-point space time intensity correlation function at 507 K. (b) $g_4(\Delta r)$ with Δt fixed at relaxation time τ calculated from $g_2(t)$. The solid line is the exponential fit to the spatial decay of $g_4(\Delta r)$.

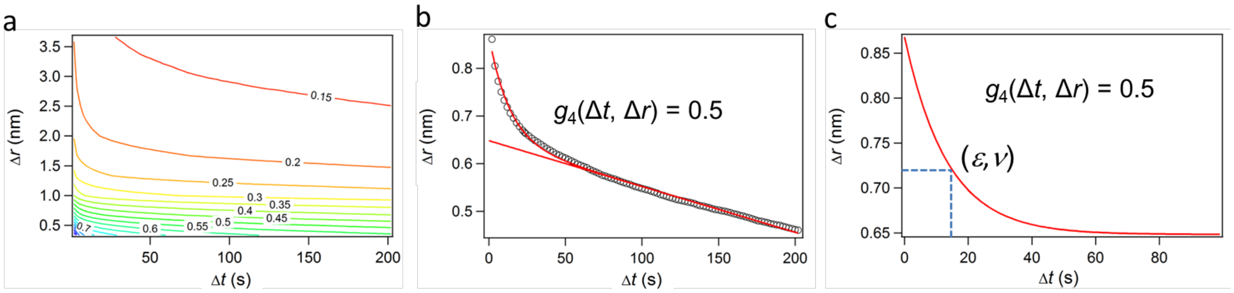

Supplementary Figure 8. (a) A contour plot of the same data in Supplementary Fig. 6a. (b) The exponential and linear fit for the $(\Delta t, \Delta r)$ contour at $g_4(\Delta t, \Delta r) = 0.5$. (c) The characteristic length ε and a characteristic time ν determined from the width of exponential fitting curve.

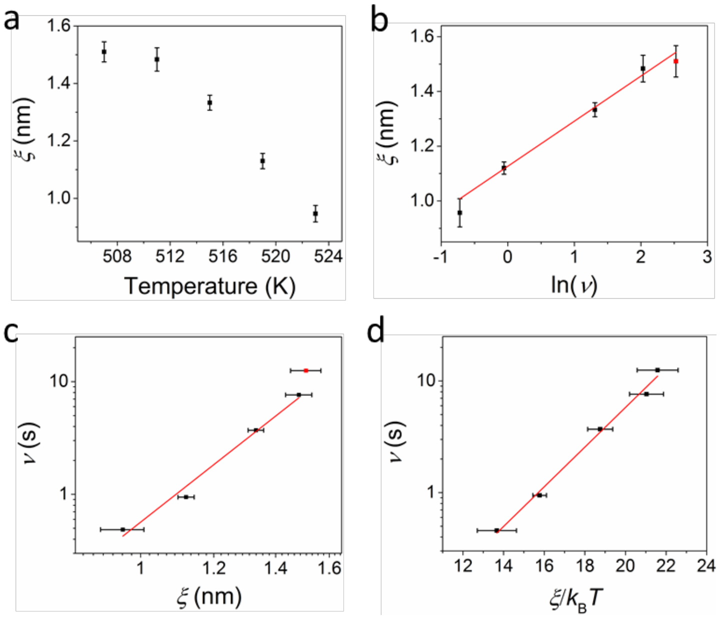

Supplementary Figure 9. (a) The correlation length ξ as a function of temperature. (b) ξ vs ln(ν), fit to Adam-Gibbs theory. (c) A log-log plot of ξ vs ν, fit to the inhomogeneous mode coupling theory. (d) A semi-log plot of ν vs ξ/k_BT, fit to the random first-order transition theory. The data are in reasonable agreement with all of the models. The error bars are the standard deviation of the mean from measurements on different nanowires.

Supplementary Notes:

Supplementary Note 1: Distribution of relaxation times and stretching exponents

Supplementary Fig. 1a shows histograms of the bulk structural relaxation time τ and β for all measured temperatures. The τ histogram $P(\tau)$ is well fit by a log-normal distribution,

$$P(\tau) = \frac{1}{\sqrt{2\pi}\sigma\tau} \mathrm{e}^{\frac{-([\ln(\tau/\tau_{\mathrm{med}})])^2}{2\sigma^2}}, \quad (1)$$

where τ_{med} is the median of relaxation time and σ controls the width of the distribution. Random, non-negative processes often follow a log-normal distribution, and similar distributions have been obtained from tracer molecule rotational diffusion measurements in *ortho*-terphenyl[1]. With decreasing temperature, the full width at half maximum (FWHM) of the histograms increases, as shown as Supplementary Fig. 1b. This increase in the spread of relaxation times is consistent with an increase in spatially heterogeneous dynamics as the temperature approaches T_g.

Supplementary Fig. 1c shows the β histograms as a function of temperature. With only a few exceptions, β is between 0 and 1.The exceptions are in some cases poor fits to the KWW form, either due to noise or due to extremely slow dynamics resulting in poorly convergence $g_2(t)$. With decreasing temperature, the β distribution shifts to smaller and smaller values. Small β is also associated with heterogeneous dynamics.

Supplementary Note 2: Temporal evolution of spatially heterogeneous dynamics

The spatial domains in the liquid state dynamics should themselves evolve with time. Wide field single molecule imaging measurements on tracer molecules in supercooled glycerol determined a persistence time for domains with relaxation time τ of ~50τ.[2] We recorded a very long data series with total data length of ~80τ_{med} was recorded with time per frame of 0.1 s at 523 K. We calculated the spatial map of τ from subsets of the time series ~20τ_{med} long, as a function of the starting time of the subset. Supplementary Movie 3 is the resulting "sliding window" movie of τ as a function of time and space. More quantitative analysis will be the subject of future work, but the movie shows that the domains with different dynamics fluctuate over time both in position on the wire and in relaxation time.

Supplementary Note 3: Data trajectory length and effect of the electron beam

ECM experiment was performed on a nanowire at 519 K with trajectory length of 2000 s and time interval of 0.25 s, ~ 40τ_{med}. Supplementary Figs. 3a and 3b show two spatial maps of relaxation time extracted from the first 20τ_{med} and second 20τ_{med} of the full data length respectively, and Supplementary Fig. 3c shows a line profile across the nanowire. The pattern of domains is not the same in the first half and second half of the data series, but the mean relaxation times are the same and the line profiles are similar. The average relaxation time from the inner bulk is 46.5 s from the first half of the series, 45 s from the second half, and 49.5 s from the correlation function calculated from the entire time series. This difference is within the ±6 s

uncertainty calculated standard deviation of the mean from several similar measurements. This comparison demonstrates first that a trajectory length of 20 τ_{med} is sufficient for our experiments, and second that there is no detectable change in the dynamics with exposure to the electron beam or to the microscope vacuum at elevated temperature.

Supplementary Note 4: Thickness dependence

All TEM experiments measure a projection of the sample along the electron beam direction, so there is the potential for the thickness of the sample to affect the results. For amorphous materials, the strong dynamical diffraction that dominates electron diffraction from crystals does not occur, but inelastic and plural elastic scattering both increase with increasing thickness. The diameter of the nanowires used for ECM measurement is ~40 nm, which is ~1.6 times the elastic mean free path for this alloy[3], so plural scattering is a concern.

Experimentally, the diameter of the nanowire has limited effect on the results over the experimentally relevant range. Supplementary Fig. 6 shows the measured relaxation time in the center of nanowires of thickness ranging from 28 to 60 nm. Within the uncertainty of measurement, there is no significant change in the relaxation time for thicknesses between 28 and 45 nm. When thickness increases to 60 nm, the relaxation time becomes shrinks by up to 30%. All of the experimental data were acquired from nanowires within the thickness range that yields constant relaxation time in Supplementary Fig. 6.

We can understand the thickness independent nature of the ECM results by considering the number of atoms that contribute to the speckle intensity at each position. Within the column approximation, each speckle in the image is determined by the atoms in a tube with diameter set by the resolution (0.7 nm for these experiments) and running through the sample thickness. That column contains ~1050 atoms, but they only contribute to the speckle intensity if they are arranged and oriented so as to create diffraction through the objective aperture superimposed on the diffraction pattern. We can estimate the likelihood of such as event by approximating the column as being comprised of randomly oriented, nanocrystalline clusters, following the model developed by Stratton and Voyles[4]. If the clusters are randomly oriented, only clusters which satisfy a Bragg condition at the particular **k** selected by the objective aperture contribute to the speckle intensity in the dark field image. We define *A* as the fraction of clusters oriented such that there is strong diffraction. For a dark-field image[4],

$$\mathrm{A} = \frac{\varphi\Delta\theta(M/2)}{4\pi} \tag{2}$$

in which $\varphi = d_{obj}/\pi d_{diff}$, $d_{obj} = 2.51\ \mathrm{nm}^{-1}$ is the diameter of objective aperture in reciprocal space, and $d_{diff} = 9.43\ \mathrm{nm}^{-1}$ is the distance of the aperture from the direct beam of the diffraction pattern. (φ is 2π for hollow cone dark field.) $\Delta\theta$ is acceptance angle around the exact Bragg condition which will still result in strong diffraction. For small clusters, $\Delta\theta$ is dominated by shape broadening of the reciprocal lattice spots, so $\Delta\theta = d_{atomic}/d$, in which d_{atomic} is the average atomic distance 0.21 nm, and d is the diameter the cluster. M is the multiplicity of a family of planes in the crystal. We d = 1 nm as an upper bound and M = 12 as a typical value for low-index planes in metallic crystals.

Under these assumptions, the fraction of randomly oriented clusters contributing to the intensity of a speckle is ~5%. Essentially, while there are many clusters in real space, their

scattering is well-separated in reciprocal space, so we measure only on a small fraction of them at any given time. If the entire column volume is occupied by clusters, there are ~40 clusters in each column, and around 2 of them contribute to each speckle in the image. However, in the liquid it seems likely that the density of regions with sufficient order to create diffraction is less than 100%, so we estimate that the number of clusters per speckle is 1-2 for most speckles most of the time. Similar arguments for smaller clusters and higher spatial resolution yield a similar contribution of ~1 cluster in Ångstrom beam electron diffraction experiments[5]. They also underlie the interpretation of XPCS[6] data in terms of localized scattering from ordered regions in polymeric and colloidal liquids (*e.g.* [7]).

The shape of the nanowire does have an effect on the results. Supplementary Fig. 4 shows the profile of the relaxation time and projected wire thickness for a nanowire measured at 523 K and a nanowire measured at 519 K. The thickness was measured using the log-ratio method applied to elastic scattering and an elastic mean free path of 25 nm.[3] At both temperatures, the relaxation time increases in the nanowire edge, following the thickness, until it reaches a plateau in the center of the wire. These results show that the there is a thickness dependence to the data for smaller thicknesses than shown in Supplementary Fig. 6. Whether this is an artifact of the ECM experiments or intrinsic to the dynamics in a constrained system will be the subject of future research. At 523K, the plateau of constant relaxation time is ~30 nm wide, but at 519 K, the plateau is only 20 nm wide. This suggests that thickness effects become stronger at lower temperature, possibly as the dynamics become more spatially heterogeneous. In all cases, mean relaxation times and four point correlation functions described as “bulk” were taken within the plateau region at the center of the nanowire.

We report in the main text the existence of a near-surface region with faster dynamics than in the bulk. The data in Supplementary Fig. 5 show that this observation is not an artifact of the small projected thickness at the edge of the nanowires. As shown in the SEM image in Supplementary Fig. 5a, some of the nanowires on the substrate have a rectangular, rather than cylindrical cross section. Supplementary Fig. 5b shows a single frame dark field image obtained from such a wire at 519 K and Supplementary Fig. 5c shows a time-averaged dark field image from the same ECM data as Supplementary Fig. 5b. The residual contrast in Supplementary Fig. 5b arises from small thickness variations. Supplementary Fig. 5d is the relaxation time map derived from the entire data series. Supplementary Fig. 5e shows a thickness and relaxation time profile across part of the wire. The fast near surface layer is preserved around the edges of the wire, even when the edges are very close in thickness to the center of the wire. Thus, it is an intrinsic phenomenon, not a geometric artifact. The dynamics inside the nanowire also are consistent with measurements on round wires, showing similar spatially heterogeneous dynamics and the same mean relaxation time within experimental uncertainty.

Supplementary Note 5: Correlation length from the four point correlation function

Supplementary Fig. 7a shows a typical four point correlation $g_4(\Delta t, \Delta r)$ calculated from the inner bulk of nanowire at 507 K. Supplementary Fig. 7b shows $g_4(\Delta t = \tau, \Delta r)$, where τ is the average relaxation time calculated from $g_2(t)$ from the same dataset. For this example, $\Delta t = \tau =$ 320 s. We fit $g_4(\Delta t = \tau, \Delta r)$ to Supplementary Eq. (3)

$$g_4(\Delta r) = Aexp\left[-\frac{\Delta r}{R}\right] + B, \tag{3}$$

to obtain the correlation length $\xi = 2R$ associated with relaxation time τ. (R is a radius and characteristic length is usually thought of as a diameter.) The linear background is an artifact of the geometry of the data, in which we have data in two dimensions x, and y in the image, but not in z along the beam direction. The fitting in Supplementary Fig. 7b results in a correlation length $\xi \sim 1.42 \pm 0.02$ nm. Same fitting method was applied to all the other temperatures to obtain the results in the main text Fig. 4.

Supplementary Note 6: Characteristic length and time extracted simultaneously from g_4

An alternate approach to analyzing $g_4(\Delta t, \Delta r)$ is to account simultaneously for the decay in both dimensions and by doing so to extract both a characteristic length and a characteristic time from a single function. This approach is useful for our data because the functional form of $g_4(\Delta t, \Delta r)$ in Eq. (3) in the main text does not reduce to $g_2(t)$ for $\Delta r = 0$, so τ derived from $g_2(t)$ is not necessarily the most appropriate time scale.

Supplementary Fig. 8a is a contour plot of the same data in Supplementary Fig. 7a, and Supplementary Fig. 8b shows the 0.5 contour. The contour in Supplementary Fig. 8b is fit to the sum of a decaying exponential and a linear background. ξ (twice the characteristic length ε) and ν are determined by the width of exponential part, as shown in Supplementary Fig. 8c, resulting in this case in $\xi \sim 1.44$ nm and $\nu \sim 14$ s. ν derived in this method is not the same as τ derived from $g_2(t)$, so the similar of ξ derived by the two methods shows that ξ is depends on very weakly on Δt.

Supplementary Fig. 9a shows that $\xi(T)$ derived in this way increases from ~0.95 nm at 523 K to ~1.5 nm at 507 K, which is around 0.1 nm systematically higher than results derived using the method in Supplementary Fig. 7 and presented in the main text in Fig. 4. However, the temperature dependence is very similar. Supplementary Figs. 9b, 9c, 9d shows correlation between ξ with ν, fitted based on the Adam-Gibbs theory, the inhomogeneous mode coupling theory (IMCT) and the random first order transition (RFOT) theory. Reduced χ^2 is similar for the fits to all the models.

Supplementary References

1. Kaufman, L. J. Heterogeneity in Single-Molecule Observables in the Study of Supercooled Liquids. *Annu. Rev. Phys. Chem.* **64,** 177–200 (2013).
2. Mackowiak, S. A., Hermana, T. K. & Kaufmanb, L. J. Spatial and temporal heterogeneity in supercooled glycerol: Evidence from wide field single molecule imaging. *Journal of Chemical Physics* **131**, 244513 (2010).
3. Zhang, P., Wang, Z., Perepezko, J. H. & Voyles, P. M. Elastic and inelastic mean free paths of 200 keV electrons in metallic glasses. *Ultramicroscopy* **171,** 89–95 (2016).
4. Stratton, W. G. & Voyles, P. M. A phenomenological model of fluctuation electron microscopy for a nanocrystal/amorphous composite. *Ultramicroscopy* **108,** 727–736 (2008).
5. Hirata, A. & Chen, M. Angstrom-beam electron diffraction of amorphous materials. *J. Non. Cryst. Solids* **383,** 52–58 (2014).
6. Ruta, B. *et al.* Atomic-Scale Relaxation Dynamics and Aging in a Metallic Glass Probed by X-Ray Photon Correlation Spectroscopy. *Phys. Rev. Lett.* **109,** 165701 (2012).
7. Wochner, P. *et al.* X-ray cross correlation analysis uncovers hidden local symmetries in disordered matter. *Proc. Natl. Acad. Sci. U. S. A.* **106,** 11511–11514 (2009).
